# Factors affecting implementation of hospital inpatient-level care at home: a qualitative study of virtual wards in North West England

**DOI:** 10.1136/bmjopen-2025-111868

**Published:** 2026-04-24

**Authors:** Fay Bradley, Kelly Howells, Norina Gasteiger, Caroline Sanders, Thomas Blakeman, Dawn Dowding

**Affiliations:** 1 Division of Nursing, Midwifery and Social Work, The University of Manchester, Manchester, UK; 2 National Institute for Health and Care Research Applied Research Collaboration Greater Manchester, The University of Manchester, Manchester, UK; 3 Division of Population Health, Health Services Research and Primary Care, The University of Manchester, Manchester, UK; 4 National Institute for Health and Care Research Greater Manchester Patient Safety Research Collaboration, The University of Manchester, Manchester, UK

**Keywords:** Telemedicine, Health Services, Implementation Science, QUALITATIVE RESEARCH, Digital Technology, Hospital to Home Transition

## Abstract

**Abstract:**

**Objectives:**

To identify key factors influencing the implementation of technology-enabled virtual wards (VWs), also known as hospital at home, drawing on the qualitative accounts of stakeholders involved in implementation, using the updated Consolidated Framework for Implementation Research (CFIR) as a guiding analytical framework.

**Design:**

Qualitative semi-structured interviews with implementation leads. All interviews were conducted online, using MS Teams or Zoom, between January–June 2024, and audio-recorded with consent. Audio-recordings were transcribed, anonymised and exported to NVivo V.12 Pro software for data management. The updated CFIR was used to guide thematic analysis of interview data.

**Setting:**

Adult VW services in one regional health and social care system in North West England, UK.

**Participants:**

Service implementation leads from 11 hospital sites providing adult VW services. Job titles and roles varied across sites and included both operational and clinical service leads.

**Results:**

20 interviews were conducted with 22 participants. Four implementation themes were identified: (1) complexity and adaptability: the ability to adapt the service to local conditions was valued by leads, but also contributed to wide variation in operational, clinical, workforce and digital components of VW models; (2) resource and infrastructure: workforce capacity was identified as a key implementation challenge along with information technology system capability and interoperability; (3) performance demands: leads were concerned that an excessive focus on bed numbers and occupancy levels, without accounting for patient acuity, could negatively affect implementation, straining the service and staff capacity; and (4) readiness for change: organisational and professional readiness for change was considered crucial for increasing referrals and enabling successful implementation, yet leads reported that the level of behavioural and cultural change required had been underestimated.

**Conclusions:**

Implementation of a national VWs programme has resulted in wide service variation in one UK region, which raises questions about service equity and poses challenges for wider programme evaluation. Despite this variation, common factors found to help or hinder implementation have been identified. This study provides greater understanding of the factors that influence the implementation of VW services and outlines actionable insights to help refine VW strategies. These insights can support future planning and sustainability of technology-enabled inpatient-level care at home more widely.

**STRENGTHS AND LIMITATIONS OF THIS STUDY**

- Qualitative design to capture in-depth information on the experiences of implementing virtual ward (VW) services.
- Study sample included all sites providing adult VW services across one region to enable comparative analysis of implementation approaches.
- Analysis informed by an established implementation framework, the Consolidated Framework for Implementation Research.
- The focus on one regional health system means that the organisational context could determine the implementation processes involved.

## Introduction

 Virtual wards (VWs), often also referred to as Hospital at Home (HaH), have emerged as a key component of wider healthcare system strategies to address overwhelming demand on hospital inpatient and urgent and emergency care. These services aim to provide acute clinical services to patients in their usual place of residence, equivalent to that received in acute inpatient hospital care. They may be used to prevent the patient being admitted into hospital (‘step-up’ approach) or to support early discharge from hospital (‘step-down’ approach). Various forms of VWs/HaH models have been in operation for several years in the UK and other countries.^1^ However, in April 2022, the National Health Service (NHS) in England introduced a large-scale national VW programme, with an allocation of up to £450 million of funding.^2^ Key components of the NHS England (NHSE) model of VWs include: clinical oversight from a consultant physician/practitioner or general practitioner (GP); technology-enabled care, including remote monitoring; hospital-level diagnostics and intervention/treatment; time-limited with a recommended length of stay up to 14 days; and intended for acute care not safety netting or intermediate care.^3^ Throughout this paper we use the term VW to refer to both VW and HaH services.

Through the NHSE programme each geographical health and social care organisation in England, known as an Integrated Care System (ICS), was asked to introduce new VWs or expand existing models. ICSs are partnerships with collective responsibility for planning services for a geographical area. There are 42 ICSs in England, each led by an Integrated Care Board (ICB), which is the statutory body responsible for planning NHS services for that area. ICBs were set a target of delivering 40–50 VW ‘beds’ per 100 000 patient population by December 2023,^2^ initially focused on acute respiratory infection and frailty pathways, followed by a heart failure pathway.^3^ Although VW services have expanded rapidly, as of December 2025, the target of 40–50 VW beds per 100 000 population has not been reached by the majority of ICBs, with a national average capacity of 20 beds per 1 00 000 patients. The average occupancy of VWs in England is around 80%.^4^

The provision of inpatient-level care at home is growing across healthcare systems. Current evidence, although inconsistent or of low-medium certainty,^5^ suggests that these models are acceptable to patients^6^ and can deliver comparable outcomes to inpatient hospital care.^7^,^9^ Evidence specific to technology-enabled models of care is more limited^10^ and research exploring the factors that influence their implementation is scarce.^11 12^ Implementing complex interventions at scale, rapidly across different settings and contexts is inherently challenging.^13^ Incorporating digital health technologies adds further complexity and introduces additional implementation barriers.^14^ Although literature on VW services is growing, findings often relate to HaH model services, which traditionally involve more in-person care, with less reliance on technology than VWs.^11^ Studies that offer some insight into the implementation of VWs suggest that both clinician reluctance to refer to the service^15 16^ and technology issues, including poor interoperability of systems and limited user acceptance of remote monitoring, can hinder implementation.^5 12^ However, the need for further research on the implementation challenges of VWs has been established.^11 12^ Greater understanding of factors influencing implementation is essential to refine current strategies and support future planning and sustainability of technology-enabled inpatient-level care at home more widely. The aim of this paper was to identify key factors influencing implementation of VWs, drawing on the qualitative accounts of stakeholders involved in implementation, using the updated Consolidated Framework for Implementation Research (CFIR) as a guiding analytical framework.^17^

## Methods

The data presented in this paper are part of a larger evaluation of the VW programme in one region of North West England, which included several components and mixed methods. This paper draws on one qualitative component of the study, involving semi-structured interviews with VW service and implementation leads from across the 11 hospital sites providing adult VW services in this area, and was reported using the Standards for Reporting Qualitative Research^18^ (see online supplemental file 1).

### Sample and setting

Four clinical networks were established to implement the VW programme across the region, offering hospitals the opportunity to ‘buddy up’ to support with implementation. The region covers the largest patient population in England and, during the study period, had the largest VW programme. At the point of data collection, services across sites varied in their maturity, ranging from 10 to 24 months since implementation.

Contact details for clinical and/or operational leads involved in the implementation of VWs were provided by the regional Health Innovation Network; additional contacts were identified by participants through snowball sampling. All participants were provided with a Participant Information Sheet and gave either their verbal or written consent to take part.

Interviews were semi-structured using topic guides informed by existing HaH/VW and Implementation Science literature. All interviews were conducted online, using MS Teams or Zoom, between January–June 2024, by four experienced qualitative health services researchers. Interviews lasted between 35 and 75 min and were audio-recorded with consent.

Interviews explored topics including: the key components of the adopted VW model(s); processes for referrals, eligibility assessments and admissions; integration at system level; use and acceptability of technology; workforce and capacity; and views on implementation and evaluation of the service. See online supplemental file 1 for the interview schedule.

#### Analysis

**Figure 1:**
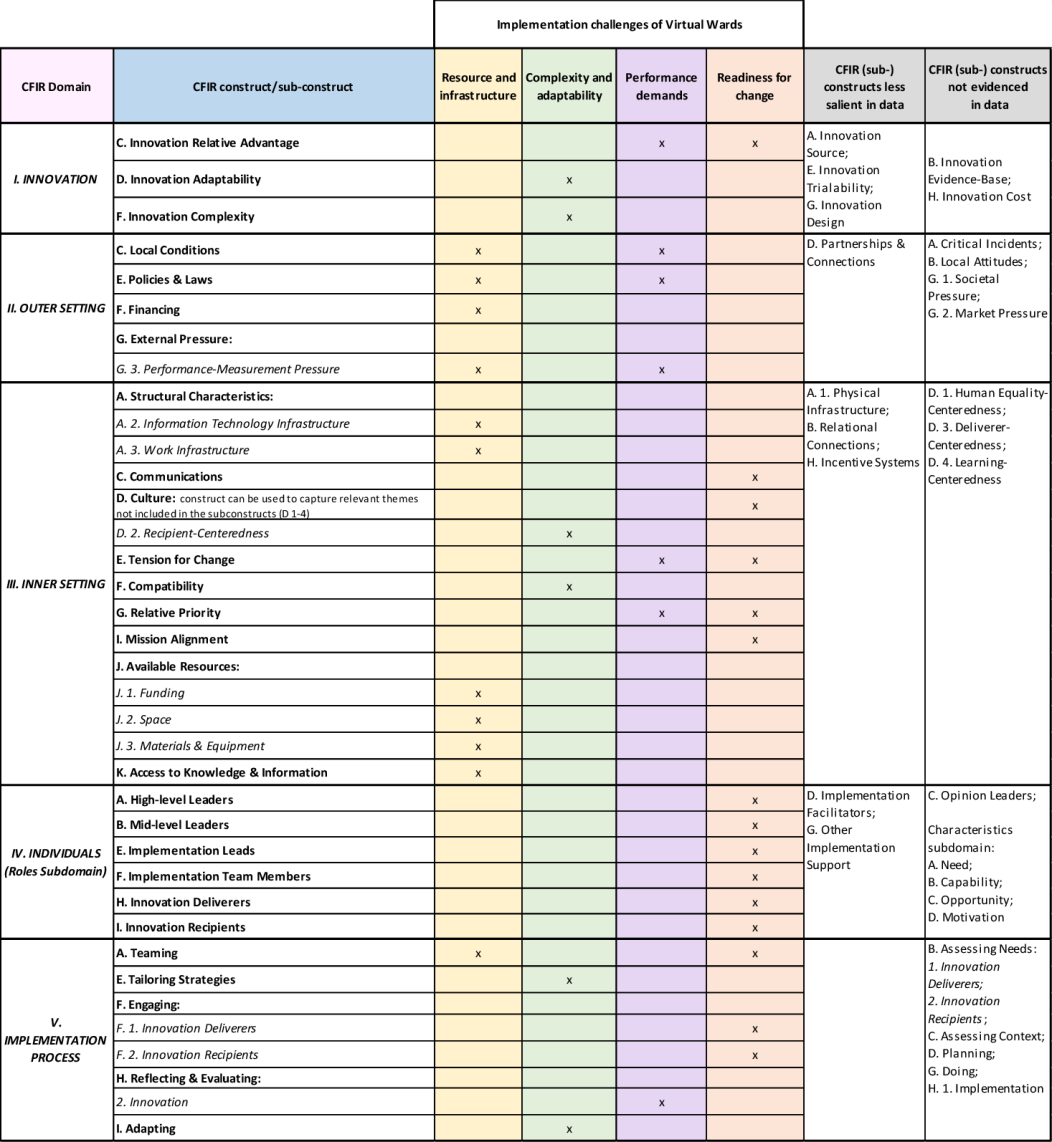
Mapping of CFIR constructs to virtual ward implementation themes. CFIR, Consolidated Framework for Implementation Research.

Audio-recordings were transcribed manually (by a professional transcription company), anonymised and exported to NVivo V.12 Pro software for data management (QSR International 2018). The updated CFIR^17^ was used to guide thematic analysis of interview data. The CFIR is a framework of constructs developed to help understand barriers to and facilitators of implementation and organised around five main domains: innovation, outer setting, inner setting, individuals and implementation process. Preliminary coding combined deductive and inductive approaches. Deductive codes based on the topic guide were applied, with additional researcher-generated codes developed inductively from the data. Following preliminary coding, secondary coding was applied using a deductive coding template based on the CFIR domains and their (sub-)constructs. Mapping of the preliminary codes to CFIR and the CFIR domain/constructs’ saliency to the interview data was noted throughout, along with any constructs that were not evident in the data (figure 1). Codes were clustered according to the most salient CFIR constructs and developed into broader cross-cutting themes, capturing the key insights. Coding templates were agreed across team members, and a subsample of transcripts were double-coded to support consistency. Coding discrepancies were discussed and resolved during weekly analysis meetings, which helped to support coding, interpretation and analytical approach. The research team met regularly throughout data collection and analysis and once deemed to be theoretically sufficient,^19^ data collection was closed. The research team are experienced qualitative researchers with expertise related to digital health evaluation and inequalities, patient safety, health service organisation and clinical decision-making. None of the research team had pre-existing relationships with participants, nor were involved in the implementation or provision of VWs. One author is a GP who has exposure to VWs in their clinical role, although in a different region in the UK. This study was conducted as an independent evaluation.

### Patient and public involvement statement

To ensure that this study focused on topics pertinent to both the healthcare system, patients and carers, we first held two workshops with health and social care leaders and stakeholders, and the National Institute for Health and Social Care Applied Research Collaboration Greater Manchester Public and Community Involvement and Engagement Panel. Additionally, to ensure wider community engagement, four workshops were held with diverse community groups. Two of these were with non-English speaking communities in partnership with community-based organisations.

## Results

20 interviews were conducted with 22 participants, across 11 sites; 2 participants held network level roles, spanning several sites. All participants were involved in the implementation of VWs in their area and can be categorised into two groups: (1) clinical VW leads (n=12), primarily leading or clinically overseeing the service; and (2) operational managers (n=9), responsible for VWs as part of their portfolio. One participant held a strategic/transformation role responsible for planning and coordinating VWs at organisational level. Job titles and roles within these categories varied across sites and are not presented to preserve anonymity. At least one clinical and one operational participant was interviewed at each site, apart from four sites where only a clinical representative was available. 35 individuals were approached initially, with 15 not taking part. The majority of those declining suggested alternative individuals who they believed to be better placed to participate due to experience (all of whom were subsequently approached and agreed to participate). Five individuals did not respond to the invitation. Those who declined were mostly concentrated in roles that were either operational, strategic/transformation or a hybrid of both. Illustrative quotes from participants are presented for each theme.

### Implementation challenges

Four themes were identified: (1) complexity and adaptability, (2) resource and infrastructure, (3) performance demands and (4) readiness for change. See figure 1 for mapping of these to CFIR constructs.

#### Complexity and adaptability

The NHSE VW programme represents a complex intervention involving multiple stakeholders, teams and systems across varied implementation sites. Leads valued the ability to adapt services to local conditions, which enabled them to build on or modify existing services to meet VW requirements and deliver these at pace:

…we were using that [technology provider] previously for telehealth monitoring for 150 patients with long-term conditions…So, when we moved towards virtual wards we’ve just expanded our number of kits…So therefore, all of the staff knew about it and how to use it, and how to implement it already, because we’d been using it for 3 years already. Int 12, Site 2

For a care org[anisation] that weren’t delivering anything before, it’s harder for them to start it cause they’ve not got the existing service that they can expand to deliver something more. We’ve got a service that they can take from the stack, it’s just about increasing the acuity of what they’re taking. Int 21, Site 11

Conversely, others suggested that although slower to implement, designing services from scratch ensured better alignment with the NHSE VW criteria and reduced overlap and/or duplication with existing services:

If you’re managing a long-term condition on a virtual ward, how is this new activity going to be different from that long-term condition management? Because it was very much new activity of patients that otherwise would be in a hospital bed…I think it’s probably more difficult to not start from scratch and try and build on existing services there that might not necessarily be being used in the way we had to use it for the purpose of the [area] programme. Int 06, Network D

Linked to this was widespread concern among leads that the VW criteria (developed by NHSE at the time) were too vague and had resulted in wide variation in interpretation across sites:

I’m not sure we’ve nailed down what the actual criteria is still. So, even though it keeps being told, well this has to be a patient that would otherwise be in hospital, they have to be under the care of a consultant, you know, and they have to be having daily ward rounds like they would in hospital, that is clear, but the interpretation of that is very different in different areas. Int 05, Site 3

… I think people are all at different stages in their delivery of their services… I think part of the problem is, we’ve revisited the inclusion for virtual ward that many times as a locality across [the region] that it’s dead clear that we still all don’t think the same, everyone is probably going to be doing differently. Int 21, Site 11

**Figure 2:**
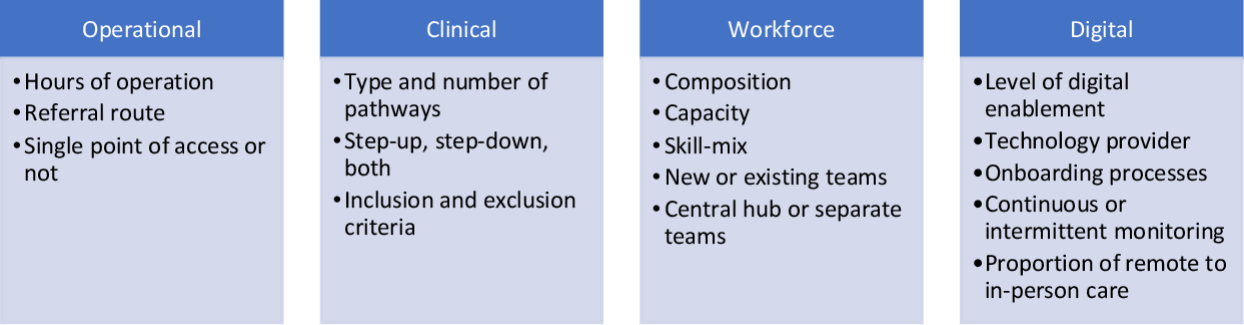
Key elements of variation in virtual ward implementation across sites.

VW models varied widely across the area, with differences evident in four key elements: operational, clinical, workforce and digital (see figure 2). Operational differences were seen in the hours of operation, referral routes to the service and availability of a single point of access. Clinical differences were apparent in relation to the type and number of clinical pathways implemented at sites, the patient inclusion/exclusion criteria adopted and whether sites offered step-up, step-down services or both. Workforce models differed widely across sites in terms of size and skill-mix. At some sites, dedicated VW teams were established, while others expanded existing community teams. Several sites adopted a hub-style model delivered through a single team, whereas others used separate teams with different points of access, depending on the clinical pathway.

Digital variation was evident, with the use of three different technology providers across the region. There was variation in the level of monitoring offered with some sites opting for continuous monitoring, while others chose intermittent spot monitoring. The level of technology use also varied widely between sites, ranging from full remote monitoring to hybrid remote and in-person care approaches. One site had stopped using remote monitoring and was operating solely with in-person care. Technology use also varied within sites depending on the condition pathway and patient needs, with technology used least for frailty patients.

#### Resource and infrastructure

Resource and infrastructure, in the form of existing services and workforce, posed both opportunities and challenges for VW implementation. As described above, some sites built on existing services, enhancing existing workforce with additional GP and/or consultant medical support (a requirement of the NHSE VW programme framework), whereas others established new dedicated VW teams. Both approaches brought challenges: new teams faced recruitment difficulties/delays and needed time to embed, while existing teams struggled to adapt to new models of practice and technologies. In some cases, a number of separate teams were involved in VW provision at a site, often working in silos and sometimes governed by different organisations:

But I guess one of the main barriers…is the fact that we were building on existing services. So we’ve got teams that are working very much in silo…From a learning point of view, that’s something that we’re going to look to develop with recruitment and funding for next year, is more of a model structure, because working in silos and working with separate teams, didn’t lend itself very well to pursue the remote monitoring. Int 08, Site 8

…Having staff that are running off different policies is also a nightmare when managing staff. You’ve got one staff member that can do one thing and you’ve got one staff member that can’t, and that causes issues. Int 09, Site 9

At the moment, they are run as two separate services but under one umbrella, we’re working much more closely to bringing them together as one…team. Int 20, Site 6

Recruitment of VW staff was reported to be hampered by short-term funding cycles and fixed-term contracts, which were not considered an attractive option to staff. Across sites, there was consensus that more advanced clinical practitioners (ACPs) were needed due to their advanced clinical skills and autonomous practice, yet this role was in high demand and in short supply across the NHS:

…there’s not a lot of ACPs out there and we’ve gone out for secondment positions, they’re not substantive yet because the funding hasn’t been agreed. Int 19, Site 4

Some leads reported that the skill-mix and capacity of their VW teams was appropriate; others were reviewing their staffing models. Several sites reported that they were unable to increase bed numbers to meet the prescribed VW bed targets, due to staff recruitment issues. Others reported needing to delay patient onboarding due to lack of workforce capacity, especially if in-person care was required:

…we struggled with the recruitment [of virtual ward staff]…we’re never getting above a certain threshold with the staffing to grow the beds. And that meant that the numbers we envisaged for step down for frailty are still less than what we would want them to be. Int 07, Site 11

So sometimes there’s not been the capacity to care for that patient because sometimes it can hinder us onboarding them, and capacity for us to go out and do that face to face. Because even though we can monitor virtually, we still need to go out and give the education and the training around that to see if it’s suitable. Int 09, Site 9

Capacity to onboard patients was influenced by levels of patient acuity, which fluctuated across and within sites. It was felt that additional VW funding for workforce was essential to maintain consistent safe staffing levels:

…it almost feels like there needs to be a RAG [Red-Amber-Green] rating in terms of how many you can have of each, so you couldn’t have 26 ambers and 15 reds with one individual. But it has made us look again at our medical model; we’re just reviewing that now. And that’s, I think, where the additional funding needs to be given, because you wouldn’t run an acute ward on the same staffing levels. Int 10, Site 5

Existing information technology (IT) infrastructure within NHS organisations was also viewed as a significant challenge to effective implementation. Variation in electronic patient records systems and other IT platforms hindered integration of VWs with other services and limited opportunities for cross-site/network collaboration. The capabilities and interoperability of these systems differed widely. Some leads reported that the continued use of outdated systems prevented further development and expansion of the VW programme. Others faced difficulties joining up services and evaluating impacts due to some services still being paper-based:

I think we’ve got some antiquated systems [at some of the localities in the Network]…that again may have stifled some opportunities to explore technology working differently out with patients. Int 06, Network D

IT was a barrier as well, because we’ve got different patient records systems. So there’s still just lots of practicalities that you wouldn’t think of to overcome. Int 07, Site 11

I think data as a whole has been a big challenge, and I think one of the challenges across our locality has been that some of our services are still on paper, so we’re not a paperless system. Int 08, Site 8

### Performance demands

Participants across sites were critical of the regional focus on reporting VW bed numbers and occupancy levels, which they felt to be excessive. Leads suggested shifting performance measures away from activity and throughput towards patient outcomes and experience, inclusion, acuity levels, costs and wider NHS and social care system impacts. There was consensus that sites were recording and reporting VW numbers differently and concern that existing services were included within VW numbers at some sites. As such, participants felt that comparisons of bed numbers and occupancy levels across sites was unhelpful and not comparing ‘like for like’:

…why is it that [other sites] they’re getting all them patients on [virtual wards] and we’re not? …Is it us that are not counting the same, are we not comparing oranges with oranges?…So, if I’m not counting the same as what everybody else is counting, and therefore it looks like we’re not providing a value for money service that’s efficient and effective…I could go out tomorrow and say to urgent community response, right, I want all your numbers, all your IV patients, and I’m going to put them all on. It’s not morally right. It doesn’t sit right for me because that’s not what we should be doing. But if I have to do that to secure my funding then I will relook at it again. But then I’ve got an internal conflict because it just doesn’t sit comfortably with me. Int 05, Site 3

You’ve got to be careful and we’re always careful, you can’t just count stuff that you’re already doing as virtual wards. We’re really mindful of that as well. Int 21, Site 11

Participants felt that a focus on occupancy levels, without consideration for differences in patient acuity levels, contributed to ‘unfair’ comparisons being made between sites. High proportions of higher acuity patients intensified staff workload and reduced capacity to increase occupancy levels:

I’d like to see across [the region] what is the percentage of greens, ambers and reds [acuity levels of patients] that people are carrying, if you like? Because if you’ve got 70 per cent of your activity is green then that doesn’t incur all of those senior clinical reviews, it doesn’t incur going out to see the patients, you know…Whereas if we’ve had our service where we’ve had 26 ambers at one point, that takes a lot of resource, then, doesn’t it, and it takes a lot of time, energy, to keep that person out of hospital. Int 10, Site 5

The pressure to increase VW numbers was felt intensely across sites. Leads expressed concern that this expectation could dilute the service, resulting in less acute patients–who may not have required an inpatient stay–being admitted to increase occupancy, while maintaining manageable workloads. Others were concerned that expanding bed numbers could overstretch the workforce to a level at which safe patient care could no longer be assured:

It’s all well and good saying we need 70 patients on our books by April, if that’s the case, are they going to be appropriate patients? Are they going to be ones that we’ve just onboarded because they say we have to have these numbers in? And then if they are acutely unwell or they’re really frail, can we safely deliver that? Int 09, Site 9

### Readiness for change

The importance of organisational and professional readiness for change was cited by leads as a key factor influencing the successful implementation and delivery of the VW programme. Leads from sites with more established VW services (in terms of maturity) identified the importance of early high level Executive Board support, along with strong clinical leadership:

So, we had executive board backing and approval and it was a huge part of the strategy for not only the coming year that we’ve just worked through but within their three to five year plan. Which I think is hugely important to anybody entering into this venture, they need that level of backing at the executive board level. Int 14, Site 1

…having that strong, clinical leadership is really key, and you can [implement] quicker, faster…and if you’ve got a clinician that’s not bought into it, then it’s going to be very difficult. Int 12, Site 2

Sites where this high-level support had not been secured or had not been secured early in the implementation process had faced difficulties with staff engagement and referral to the service:

…this was a transformation programme that was led by the community with very little senior involvement from the acute sites, just us as site leads. The dynamics and the way of set up and support and views, and things, would have been better supported if everyone was involved from the beginning at those senior levels. Int 16, Site 6

Linked to this was the frequently cited barrier of culture and lack of readiness for change. Leads commonly reported that the VW model challenged a prevailing culture that hospital care was the safest option for patients and often faced opposition from staff regarding referral into the service, particularly from consultants:

… some of the consultants are, well how do I know that what you’re doing is safe, because there’s not been any data to say that it’s safe. So if you go and speak to the cardiologists, where they’re used to dealing with evidence base and research, where is the evidence and the research that this is safe or better? Int 18, Site 7

Along with the influence of this prevailing culture, leads suggested that reluctance to engage with or refer to the service was also related to a limited understanding of who the service was for, how it operated and concerns about safety and risk:

… I think it’s not that the alternatives are not safe, I think it’s that the belief of what is better, is that hospital is the safest place. Now all the evidence would point it’s not but at the core, we built hospital, hundreds of years ago, to be a really safe place for sick patients. So, how do you change that and then you would get into the practicalities of, well I don’t know what the referral criteria are, can they look after this type of patient, who does that type of patient, what is the workforce, how often will they get reviewed…who is their consultant if they are not in the hospital because I’m not having my name as consultant if I’m not seeing them every day on a ward round. Int 20, Site 6

…with a lot of acute consultants where they like to have it close and own it and it be comfortable for them so they can see them every day, do a ward round every day and it means that they feel more comfortable that someone’s in that bed. I think it’s hard to get them away from that. It’s that relinquishing control that I think some of the acute colleagues struggle with because they still very much have that, we know best, we’re consultants, and that attitude. Yeah, I think that is a bit of a barrier. Int 21, Site 11

Several leads felt that the implementation of VW services also required elements of an accompanying behaviour change programme. On reflection they felt that this need—as well as the associated time, capacity and resource to deliver it—had been underestimated:

Barriers were the consultant body, because it was really hard initially to get consultants to refer to us onto the virtual wards, because it was a complete change in behaviour and culture to how they…it still is, they don’t like referring their patients into the virtual ward. They would rather have their patients in front of them and that idea of a new way of working is very difficult for them to understand. Int 12, Site 2

…it’s an automatic presumption that everyone will refer and buy in and they won’t, because everyone manages risk very differently… that’s my one and only criticism about how we’re setting up across our huge organisation is this has probably been one of the biggest programmes of change that we’ve ever seen and the bit that was missing was the behavioural cultural element. Int 16, Site 6

If we’re going to expand the capacity [of virtual wards], we absolutely needed a behaviour change programme that sat alongside that. I think whilst I said that, I don’t think we did it in a structured, focused, mindful way…So, key for us is Hospital at Home is a mindset that effectively changes the paradigm that we’ve had in medicine…where if people are sick, they come to hospital and that was never going to change overnight… I think we underestimate the change that this needed to support something like that and that’s where it feels like you are pushing more uphill then to try and get people to use something you’ve designed. Int 20, Site 6

This ‘missing’ step was a recurrent theme in relation to the challenges faced during implementation. Leads also stressed that as well as this initial work, there was a need for continuous communication with potential referrers about the service, including information about the aim of the service, how it might differ from other existing services, how it operated and who was eligible and suitable. To address shortfall in referrals, some sites had adopted the strategy of implementing a ‘pull’ model. This involved one or more staff members actively identifying potential patients, either by maintaining a presence on wards to remind staff about the service and discuss patient eligibility or by searching the electronic patient record system for suitable candidates.

## Discussion

This study has identified four overarching factors found to influence the implementation of VWs services, based on the accounts of stakeholders involved in implementation across one region in England. *Complexity and adaptability*: The VW programme was found to be a highly adaptable intervention, which implementation leads valued, but which also led to wide variation and complexity across sites, posing problems for network collaboration and comparative evaluation. *Resource and infrastructure*: Creating the ‘right’ workforce model and increasing staff capacity through additional recruitment on short-term funding cycles was identified as a key implementation challenge along with navigating IT systems’ lack of capability and inter-operability. *Performance demands*: Implementation leads expressed concerns that an excessive regional focus on bed numbers and occupancy levels could undermine implementation, creating pressure to onboard inappropriate patients or overstretch staff and service capacity, potentially compromising safety. *Readiness for change:* Organisational and individual readiness for change was considered crucial for increasing referrals, yet some sites reported that the need for staff behavioural and cultural change had been underestimated.

The strengths of this study include its qualitative design to capture in-depth accounts from those directly involved in the implementation of VW services. Stakeholders from each of the 11 VW sites participated, which enabled comparison across settings and identification of variation in service components. Analysis was also informed and guided by an established Implementation Science framework. Limitations include the scope of the study, which focused on one regional area, and this context could determine the impact these implementation factors had on services. However, the study focused on the largest regional VW programme in England, in an area with wide variation across sites in terms of socio-demographic characteristics and area deprivation.

This study provides insight into the factors that helped or hindered implementation of VW services as part of a nationally funded programme. A previous study examined the views of ICS commissioners in England regarding the implementation of the national VW programme, also using a CFIR informed approach.^12^ The authors report uncertainty surrounding the definition of what constitutes a VW and that guidance was being interpreted differently. Barriers to implementation that were identified included lack of interoperability of IT systems, narrowly defined parameters of success and limited timescales within which to assess impact, all of which are confirmed by our findings. The earlier study, however, captured views from commissioners around the feasibility and acceptability of implementation in the initial year of the programme, with many VWs not yet fully established nationally. Our study captured data on the second year of the programme and drew on the accounts of those directly involved in implementing the service at VW site level, allowing us to identify variation across sites and compare implementation approaches.

This study highlights substantial variation in service models and implementation approaches, despite national and regional efforts towards standardisation.^20^,^22^ This variation exemplifies the tension between system-wide standardisation and the local customisation often required in practice.^23^ A study on the implementation of remote monitoring for frailty VWs also demonstrates similar tensions, which was heightened by concern over the suitability of technology-enabled remote care for elderly frail people and the need to adapt services accordingly.^24^ In our study, while leads viewed the adaptability of the intervention as important for meeting patient and local population needs, the level of variation in service components observed across sites may affect patient equity, especially in terms of access and experience. Differences in patient criteria across sites could result in patients being eligible for VW at one site but not at another. Variation in level of monitoring (continuous or intermittent), the type of technology adopted and ratio of in-person versus remote care could lead to patients being offered substantially different levels of care depending on geographical location. Further work is needed to examine equity issues around VW care, particularly patient and carer views and experiences of accessing and receiving VW services.^25^

The NHS’ 10-year health plan for England cements the role of technology-enabled VW services in future healthcare delivery, promising further expansion of these services between 2025–2028.^26^ Our findings have implications for the future of these services as implementation strategies are key to sustainability.^27^ For VW implementers, strategies to increase referrals, maintain service visibility and staff engagement may be particularly important.^18^ Approaches such as VW staff visibility on hospital wards and at ward rounds, as well as active scoping for patients using electronic patient record systems were adopted at some sites in our study. However, most strategies were reactive measures introduced to address shortfalls in referrals and increase bed numbers in line with expected targets. VW services are transformation programmes that challenge the cultural norm that hospital is the safest place for patients and shift acute care towards greater use of remote monitoring technology. Proactive strategies to assess and address readiness for change at organisational and individual staff level were largely felt to be absent from the implementation of VW services.^28^ The inner setting’s ‘culture’ and ‘mission alignment’ must therefore be taken into consideration before and during the implementation process. The added dimension of nationally prescribed targets for bed numbers and occupancy levels was also found to have impacted on VW implementation. The introduction of healthcare targets can often lead to unintended consequences, such as measure fixation, reduced staff morale, widening of health inequities and potential misrepresentation of the system.^29 30^ Although there were no suggestions of deliberate misrepresentation, leads expressed concern about inconsistent data reporting across sites and the possibility that target pressures could lead to inappropriate patient admissions or overstretch staff capacity to unsafe levels. Further research should examine the experiences of staff delivering VWs, including decision-making around patient eligibility and views on safety and capacity.

### Actionable insights

Building on the implementation factors identified, we outline a set of actionable insights for practice, aligned to the CFIR domains. These are intended to help strengthen implementation of VWs and technology-enabled inpatient-level care at home service. These are preliminary insights and further co-production with stakeholders is required to develop a more robust framework or toolkit for practice.

1. Innovation: Address unnecessary variation across services and sites by agreeing the minimum ‘core’ components that VW models should include (eg, level of monitoring, core workforce configuration) and identifying which components can be adapted to fit local context and need. Encourage sites to keep records of the adaptations made and the rationale for these so learning can be shared.
2. Outer setting: Widen VW performance metrics to include measures of patient acuity, inclusion and equity and workforce capacity, to provide more meaningful insights into performance. Clarify reporting definitions and requirements to promote more consistent interpretation and data reporting across sites.
3. Inner setting: Review workforce skill-mix regularly to ensure safe staffing, particularly during periods of higher patient acuity. Promote a ‘one-team’ approach by bringing separate teams together through regular communication and shared learning. Review digital infrastructure across services and partner organisations to identify and address interoperability issues.
4. Individuals: Use early engagement opportunities along with targeted behaviour-change support to help address concerns about shifting care away from hospital. Provide clear and ongoing communication to clinicians about referral criteria, governance, safety processes and the role of VW services.
5. Implementation process: Secure senior-level involvement early on to ensure strong organisational support and alignment with wider priorities. Create opportunities for teams across different sites to meet regularly to share implementation challenges and successes (eg, by establishing a community of practice).

## Conclusion

This study demonstrates that the implementation of VW programmes can result in wide service variation, which could impact on service equity and pose a challenge for wider evaluation. Despite this variation, common factors found to help or hinder implementation have been identified. This study provides greater understanding of the factors that influence implementation of VW services and outlines actionable insights to help refine VW strategies. These insights can support future planning and sustainability of technology-enabled inpatient level care at home more widely.

## Supplementary material

10.1136/bmjopen-2025-111868 online supplemental file 1

## Data Availability

Data are available upon reasonable request.
