# Supplementary material for: Factors affecting implementation of hospital inpatient-level care at home: a qualitative study of virtual wards in North West England
Source: BMJ Open. 2026 Apr 24;16(4):e111868. doi: 10.1136/bmjopen-2025-111868 (PMC13110518; doi:10.1136/bmjopen-2025-111868)
Supplement: online supplemental file 1 [file bmjopen-16-4-s001.docx]

**SUPPLEMENTARY FILE 1**

**Standards for Reporting Qualitative Research - completed checklist**

| **Title and abstract** | |  |
| --- | --- | --- |
|  | **Title** - Concise description of the nature and topic of the study Identifying the study as qualitative or indicating the approach (e.g., ethnography, grounded theory) or data collection methods (e.g., interview, focus group) is recommended | P.1 |
|  | **Abstract**  - Summary of key elements of the study using the abstract format of the intended publication; typically includes background, purpose, methods, results, and conclusions | P.2 |
| **Introduction** | |  |
|  | **Problem formulation** - Description and significance of the problem/phenomenon studied; review of relevant theory and empirical work; problem statement | P.4 |
|  | **Purpose or research questio**n - Purpose of the study and specific objectives or questions | P.4 |
| **Methods** | |  |
|  | **Qualitative approach and research paradigm** - Qualitative approach (e.g., ethnography, grounded theory, case study, phenomenology, narrative research) and guiding theory if appropriate; identifying the research paradigm (e.g., postpositivist, constructivist/ interpretivist) is also recommended; rationale | P.4 |
|  | **Researcher characteristics and reflexivity** - Researchers’ characteristics that may influence the research, including personal attributes, qualifications/experience, relationship with participants, assumptions, and/or presuppositions; potential or actual interaction between researchers’ characteristics and the research questions, approach, methods, results, and/or transferability | P.6 |
|  | **Context** - Setting/site and salient contextual factors; rationale | P.4-5 |
|  | **Sampling strategy** - How and why research participants, documents, or events were selected; criteria for deciding when no further sampling was necessary (e.g., sampling saturation); rationale | P.5-6 |
|  | **Ethical issues pertaining to human subjects** - Documentation of approval by an appropriate ethics review board and participant consent, or explanation for lack thereof; other confidentiality and data security issues | P.6 |
|  | **Data collection methods** - Types of data collected; details of data collection procedures including (as appropriate) start and stop dates of data collection and analysis, iterative process, triangulation of sources/methods, and modification of procedures in response to evolving study findings; rationale | P.5-6 |
|  | **Data collection instruments and technologies** - Description of instruments (e.g., interview guides, questionnaires) and devices (e.g., audio recorders) used for data collection; if/how the instrument(s) changed over the course of the study | P.5 |
|  | **Units of study** - Number and relevant characteristics of participants, documents, or events included in the study; level of participation (could be reported in results) | P.6-7 |
|  | **Data processing** - Methods for processing data prior to and during analysis, including transcription, data entry, data management and security, verification of data integrity, data coding, and anonymization/de-identification of excerpts | P.5 |
|  | **Data analysis** - Process by which inferences, themes, etc., were identified and developed, including the researchers involved in data analysis; usually references a specific paradigm or approach; rationale | P.5 |
|  | **Techniques to enhance trustworthiness** - Techniques to enhance trustworthiness and credibility of data analysis (e.g., member checking, audit trail, triangulation); rationale | P5-6 |
| **Results/findings** | |  |
|  | **Synthesis and interpretation** - Main findings (e.g., interpretations, inferences, and themes); might include development of a theory or model, or integration with prior research or theory | P.7-14 |
|  | **Links to empirical data** - Evidence (e.g., quotes, field notes, text excerpts, photographs) to substantiate analytic findings | Throughout P.7-14 |
| **Discussion** | |  |
|  | **Integration with prior work, implications, transferability, and contribution(s) to the field -** Short summary of main findings; explanation of how findings and conclusions connect to, support, elaborate on, or challenge conclusions of earlier scholarship; discussion of scope of application/generalizability; identification of unique contribution(s) to scholarship in a discipline or field | P.15-17 |
|  | **Limitations** - Trustworthiness and limitations of findings | P.15 |
| **Other** | |  |
|  | **Conflicts of interest** - Potential sources of influence or perceived influence on study conduct and conclusions; how these were managed | P.6  P.21 |
|  | **Funding** - Sources of funding and other support; role of funders in data collection, interpretation, and reporting | P.20 |

From: O'Brien BC, Harris IB, Beckman TJ, Reed DA, Cook DA. Standards for reporting qualitative research: a synthesis of recommendations. Acad Med. 2014;89(9):1245-1251

**SUPPLEMENTARY FILE 2**

# Interview topic guide

## Work package 1: Service/implementation leads

### Background

- What is your current role?
- When and how did you first get involved with the Virtual Wards (VWs) service?

### Virtual Ward models

- What VW pathways are currently operating in [locality/Trust site]?
- Are any other pathways planned? When are these likely to be operational?
- Ask for overview of pathways: conditions included; referral routes; hours of operation; extent of technology use; technology platforms; clinical team composition
- How were these/future pathways decided upon?

### Integration

- How do these models compare with national and GM developed pathways? Were any changes/adaptions needed for [site]? Why was that?
- Have models been shared or co-developed with other localities in the VW network or elsewhere?
- Are VW care models and pathways integrated at ICS and network board levels?
- What do you think about the VW network model? What are the opportunities and challenges?
- To what extent are integrated systems required for VW delivery?

### Referral, assessment and admission processes

- At [site], what are the referral routes for VWs? Who is identifying patients and where does this happen?
- Once referred, how are patients assessed for suitability for VW care? What criteria are being used/how developed? Who makes this assessment?
- How is it decided as to whether it is safe for a patient to be in a VW?
- Patient acceptability - Do you have a sense of what factors might influence whether a patient accepts or declines VW care? What is the role of carers in this decision?
- Are there certain patient groups that tend to accept/decline more than others? Why do you think this is?

### Technology – onboarding and guidance

- Which technology provider are you using at [site]? Ask for overview of the key functions.
- Why was that technology provider chosen? What do you think are the benefits/drawbacks of that system?
- What training or support did staff initially receive? Who delivered this? How is ongoing support provided? Were there any challenges when initially learning to use the system?
- How is the technology onboarding process carried out with patients? Who provides training and advice on the equipment and monitoring? How is ongoing technical support provided?
- How and when are decisions made about patient/carer ability to use the technology?
- What happens if a patient or carer is unable to use the technology?

### Organisation of care

- How is the care team organised that provides care in the VW?
- Who makes up the team? Are these dedicated VW staff or redeployed from elsewhere?
- Do you have enough capacity in the team currently? Are there plans to increase capacity? Any workforce challenges?
- How and how often are patients monitored, and who by?
- What happens if there are patient safety concerns?
- How do clinicians identify if a patient requires further input or intervention (identifying deterioration)? What do they do? What decisions do they take on the basis of that assessment of need?

### Evaluation

- What do you feel is currently working well with the VW services? What is not working so well and needs to be improved?
- What do you think has helped the implementation of VWs at [site] What are the key barriers? *[implementation factors e.g., availability of resources, existing integration/networks/relationships/, IT systems, patient acceptability, internal/external policies)*
- Any thoughts on how the VW programme has been delivered at national level? At GM level? Has this helped/hindered progress at [site]?
- How are you evaluating the VWs service at [site]? What outcomes are you looking at? What does/should a successful VWs service look like?
- Anything else you would like to add?
