# Supplementary material for: Factors affecting implementation of hospital inpatient-level care at home: a qualitative study of virtual wards in North West England
Source: BMJ Open. 2026 Apr 24;16(4):e111868. doi: 10.1136/bmjopen-2025-111868 (PMC13110518; doi:10.1136/bmjopen-2025-111868)
Supplement: Supplementary file 2 [file bmjopen-2025-111868.reviewer_comments.pdf]

PEER REVIEW HISTORY

BMJ Open publishes all reviews undertaken for accepted manuscripts. Reviewers are asked to complete a checklist review form and are provided with free text boxes to elaborate on their assessment. These free text comments are reproduced below.

ARTICLE DETAILS

Title (Provisional)

Factors affecting implementation of hospital inpatient-level care at home: A qualitative study of virtual wards in Northwest England.

Authors

Bradley, Fay; Howells, Kelly; Gasteiger, Norina; Sanders, Caroline; Blakeman, Thomas; Dowding, Dawn

---

VERSION 1 - REVIEW

**Reviewer**	**1**
**Name**	**Greenup, Edwin Phillip**
**Affiliation**	**Queensland Health, Clinical Excellence Queensland**
**Date**	**28-Nov-2025**
**COI**	**None**

Thank you for this submission. I would be interested to learn a little more about the process of your analysis, including whether audio-recordings were manually or automatically transcribed, but otherwise this article makes a good contribution to the literature on the implementation of virtual wards/hospital-at-home services.

**Reviewer**	**2**
**Name**	**Chandra, Saurabh**
**Affiliation**	**Center for Telehealth, University of Mississippi Medical Center**
**Date**	**09-Dec-2025**
**COI**	**None**

Virtual Wards or Hospital at Home programs offer unique opportunities for patients to be safely managed in their home settings for acute conditions that would otherwise require them to be admitted in the hospital. These programs have proven benefits such as enhanced patient experience, lower cost of care, improved capacity of hospitals to admit sicker

patients. As such, there is a broad interest in providing evidence based best practices for successful implementation of these programs.

In this regard, the study submitted is timely. The study delineates key factors that can impact the implementation of these programs using qualitative accounts of stakeholders involved in implementation, using the updated Consolidated Framework for Implementation Research (CFIR) as a guiding analytical framework. While the descriptive insights are informative, the manuscript stops short of translating these findings into actionable guidance for future implementation. It does not offer a framework, toolkit, or strategy to help organizations navigate the identified challenges or operationalize the facilitators in practice.

Moreover, the manuscript does not elucidate how successful programs differed from less successful ones in their ability to leverage facilitators or mitigate barriers. Without a comparative analysis or clear lessons learned, readers will be left without a practical understanding of what distinguishes effective implementation from unsuccessful attempts.

For these reasons, the reviewer feels that the current contribution, while interesting does not yet provide sufficient depth, practical value, or generalizable guidance to merit publication in its present form. A more robust, implementation-oriented framework and clearer differentiation between successful and less successful programs would significantly strengthen the manuscript's impact and relevance for a broader audience.

**Reviewer**	**3**
**Name**	**McGillion, Michael**
**Affiliation**	**McMaster University, Faculty of Health Sciences**
**Date**	**15-Dec-2025**
**COI**	**None**

Thank you for the opportunity to review this important and timely paper that explores factors that affect the implementation of virtual wards. This qualitative study has an impressive scope, examining and analyzing the implementation experiences of 22 implementers across 11 hospital sites within a single integrated care system in Northwest England. There are several important strengths of this work including timeliness given the pressures implementers are under to execute virtual wards models in multiple countries, worldwide; broad site coverage across an integrated care system; as well as thoughtful application of the Consolidated Framework for Implementation Research (CFIR) to guide the analysis.

This paper represents an important contribution to the literature on virtual ward implementation but needs some revision to optimize its utility and transferability. Namely, the paper could benefit from additional details to enhance the clarity of the methods, as

well as a more concrete discussion of actionable recommendations, mapped back to the CFIR for implementers. In addition, the paper requires editing for grammar.

Some suggestions I have along these lines are as follows:

Methods:

o In terms of interview coverage across virtual ward pathways (across the 11 sites), it would be helpful to understand if program stage, in terms of maturity (e.g., new versus established virtual ward pathway), influenced the importance and relevance of certain themes generated.

o Given that specific job titles are not provided to protect anonymity (appropriately so), it would be helpful if the distribution of broader roles involved could be clearer (e.g., clinical leads, administrative director/managers, digital/IT lead, etc.). This would help readers further appreciate the breadth of perspectives driving the themes generated, while still protecting anonymity.

o It would be helpful to clarify numbers in terms of total invited to participate, total declined, and any key differences across leaders in terms of role/work sector and site type who declined so it can be understood if any perspectives common to virtual ward implementation are missing. If these data are not available, ideally this would be clarified.

o Further details should be added to clarify methods to assist with supporting consistency of coding within data analyzers on the team (e.g., were calibration meetings, or inter-coder agreement exercises used?)

o Ideally a statement or two could be added about the analysis team members' positionality in relation to virtual ward implementation and the data. For example, as any team members directly involved in implementation or related policy and to what extent?

Discussion

The discussion, as written, is helpful at a high level, but it could be enhanced with more concrete, actionable, guidance that is more linked back to the percepts of the CFRI. A suggestion for consideration is the addition of a concise, practice-oriented subsection with specific linkage back to the CFRI percepts for virtual ward developers to use.

For example, in relation to complexity and adaptability, it is argued that there is wide variation across operational and clinical elements of virtual ward implementations. So, could implementers be more explicitly guided to develop core virtual ward specifications where clear fidelity of implementation is required, versus other areas that can be more tailored and adaptable, and would it be sensible to recommend methods for tracking fidelity to core elements, as well as types of adaptations made through decision matrices or adaptation logs? Similarly, more explicit guidance could be provided in relation to the resources and infrastructure domains, based on the results (e.g. personnel onboarding procedures and

timelines, systematic documentation of skill mix and related adequacy for stable virtual ward implementation)

Writing and Grammar

There are too many acronyms, e.g. ICS and ICB. Suggest these be reduced for ease of reading.

The paper requires revision for grammar. For example, words are missing in the statement "Current evidence, although inconsistent or low-medium certainty (5), indicates that these…" Suggest that this sentence should read: Current evidence, although inconsistent or of low-to-medium certainty (5), indicates that these…" or of low to medium certainty…

This is one example; the p per requires a tightening of the grammar throughout for correct of issues such as run-on sentences.

VERSION 1 - AUTHOR RESPONSE

**Reviewer 1 comments**	
(3) Thank you for this submission. I would be interested to learn a little more about the process of your analysis, including whether audio-recordings were manually or automatically transcribed, but otherwise this article makes a good contribution to the literature on the implementation of virtual wards/hospital-at-home services.	Thank you for your comments. All interviews were transcribed manually (by a University approved transcription company). We have clarified in the methods that the transcription was manual.<br><br>Further details on process of analysis have been added (see comment 11 below)
**Reviewer 2 comments**	
(4) The study delineates key factors that can impact the implementation of these programs using qualitative accounts of stakeholders involved in implementation, using the updated Consolidated Framework for Implementation Research (CFIR) as a guiding analytical framework. While the descriptive insights are informative, the manuscript stops short of translating these findings into actionable guidance for future implementation. It does not offer a framework, toolkit, or strategy to help organizations navigate the identified challenges or operationalize the facilitators in practice.	Thank you for your comments. As stated in the manuscript "The aim of this paper was to identify key factors influencing implementation of VWs/HaH, drawing on the qualitative accounts of stakeholders involved in implementation, using the updated Consolidated Framework for Implementation Research (CFIR) as a guiding analytical framework".<br><br>Further co-production with stakeholders would be required to develop a framework/toolkit/strategy for practice and this is beyond the scope of the present study. The evaluation itself is a wider mixed methods study with several components, some of which are still ongoing, and we are currently working with NHS stakeholders to develop further guidance. What we have included in the revision, however, is a strengthening and greater focus on our preliminary recommendations for

	practice/actionable insights linked more clearly to the CFIR constructs, please see comment (13) below.
(5) Moreover, the manuscript does not elucidate how successful programs differed from less successful ones in their ability to leverage facilitators or mitigate barriers. Without a comparative analysis or clear lessons learned, readers will be left without a practical understanding of what distinguishes effective implementation from unsuccessful attempts.<br><br>(6) For these reasons, the reviewer feels that the current contribution, while interesting does not yet provide sufficient depth, practical value, or generalizable guidance to merit publication in its present form. A more robust, implementation-oriented framework and clearer differentiation between successful and less successful programs would significantly strengthen the manuscript's impact and relevance for a broader audience.	The aim of this paper was not to define successful/less successful programmes. The focus is on factors reported to have helped or hindered the set-up of these programmes, as reported by those closely involved in day-to-day implementation. The services provided were at different stages of development and maturity. Additionally, as identified in the findings, there was large variation in service model across sites, with concerns from providers about comparative evaluation, when not comparing 'like for like'. Instead, we have attempted to generate, from these data and guided by an established implementation framework, common factors across sites that are said to have been influential during the implementation of such programmes, providing insight into real-world 'lessons learnt'.<br><br>As discussed above we have outlined why a framework is inappropriate at this stage but have strengthened our recommendations for practice/actionable insights (please see comment 13).
**Reviewer 3 comments**	
(7) This paper represents an important contribution to the literature on virtual ward implementation but needs some revision to optimize its utility and transferability. Namely, the paper could benefit from additional details to enhance the clarity of the methods, as well as a more concrete discussion of actionable recommendations, mapped back to the CFIR for implementers. In addition, the paper requires editing for grammar.	Many thanks for the helpful comments – we address each below.
Methods:<br>(8) In terms of interview coverage across virtual ward pathways (across the 11 sites), it would be helpful to understand if program stage, in terms of maturity (e.g., new versus established virtual ward pathway), influenced the importance and relevance of certain themes generated.	Thank you for this comment. Services at the 11 sites were at different levels of maturity and this was not clearly explained in the paper previously. We have added a sentence to the methods explaining that the time since implementation ranged from 10-24 months (at time of interviews). We have decided not to group these or display further details regarding length of establishment as this could identify sites (those in the area know who the early and late adopters are).

	In relation to whether maturity influenced the importance and relevance of certain themes, we have added a sentence to the readiness to change section as this is where the reflections of leads from the more mature services are most apparent. Other themes were common across sites, with no apparent differences across maturity level.
(9) Given that specific job titles are not provided to protect anonymity (appropriately so), it would be helpful if the distribution of broader roles involved could be clearer (e.g., clinical leads, administrative director/managers, digital/IT lead, etc.). This would help readers further appreciate the breadth of perspectives driving the themes generated, while still protecting anonymity.	We have categorised participant roles into 2 broad categories - 1) Clinical VW leads 2) Operational managers. One participant also had a strategic/transformation role. We've provided a brief description of each in the manuscript and included numbers taking part. We have also clarified that 2 participants had 'network' level roles spanning several sites.
(10) It would be helpful to clarify numbers in terms of total invited to participate, total declined, and any key differences across leaders in terms of role/work sector and site type who declined so it can be understood if any perspectives common to virtual ward implementation are missing. If these data are not available, ideally this would be clarified.	We have added detail about those that declined to participate, including numbers and type of role. The majority of those invited but declining did so because they felt others would be better placed in terms of experience – decliners were mostly based in roles that were operational, strategic/transformation or hybrid of both
(11) Further details should be added to clarify methods to assist with supporting consistency of coding within data analyzers on the team (e.g., were calibration meetings, or inter-coder agreement exercises used?)	Thank you, we have added further details about our use of double-coding and weekly analysis meetings to ensure consistency of approach.
(12) Ideally a statement or two could be added about the analysis team members' positionality in relation to virtual ward implementation and the data. For example, as any team members directly involved in implementation or related policy and to what extent?	We have added a sentence to explain that no analysis team members or authors were involved in the implementation or provision of VW. This study was conducted as an independent evaluation. One author is a GP who has exposure to VWs in their clinical role, albeit in a different region in the UK.
(13) Discussion<br>The discussion, as written, is helpful at a high level, but it could be enhanced with more concrete, actionable, guidance that is more linked back to the percepts of the CFRI. A suggestion for consideration is the addition of a concise, practice-oriented subsection with specific linkage back to the CFRI percepts for virtual ward developers to use. For example, in relation to complexity and adaptability, it is argued that there is wide variation across operational and clinical elements of virtual ward implementations.	Thank you for this suggestion. We have included an additional section in the discussion that focuses on 'actionable insights', which is structured around the main CFIR domains and incorporates these suggestions. We also recognise that these insights offer preliminary recommendations and there is a need for a more robust co-produced guidance or framework.

So, could implementers be more explicitly guided to develop core virtual ward specifications where clear fidelity of implementation is required, versus other areas that can be more tailored and adaptable, and would it be sensible to recommend methods for tracking fidelity to core elements, as well as types of adaptations made through decision matrices or adaptation logs? Similarly, more explicit guidance could be provided in relation to the resources and infrastructure domains, based on the results (e.g. personnel onboarding procedures and timelines, systematic documentation of skill mix and related adequacy for stable virtual ward implementation)	
(14) Writing and Grammar<br>There are too many acronyms, e.g. ICS and ICB. Suggest these be reduced for ease of reading.	Thank you for pointing this out. We have removed reference to ICS and ICB throughout most of the paper, instead referring to this as 'region' or 'regional health and social care system'. Use of ICS/ICB is now limited to the 2nd paragraph in the introduction only, which outlines the context of the NHS programme and what ICBs were tasked to deliver.<br><br>Other acronyms that were used frequently were VW/HaH and CFIR. We have decided for ease of reading to use only VW. We have added a sentence to the introduction explaining that VW is used throughout to refer to both virtual wards and hospital at home. We hope this improves the flow of the manuscript.
(15) The paper requires revision for grammar. For example, words are missing in the statement "Current evidence, although inconsistent or low-medium certainty (5), indicates that these..." Suggest that this sentence should read: Current evidence, although inconsistent or of low-to-medium certainty (5), indicates that these..." or of low to medium certainty...<br>This is one example; the p per requires a tightening of the grammar throughout for correct of issues such as run-on sentences.	Thank you. We have corrected the sentence identified. We have also reviewed the whole manuscript thoroughly and revised grammatical issues, including run-on and long sentences throughout.

VERSION 2 - REVIEW

**Reviewer**	**2**
**Name**	**Chandra, Saurabh**
**Affiliation**	**Center for Telehealth, University of Mississippi Medical Center**
**Date**	**09-Apr-2026**
**COI**	

The revised version has considerably enhanced the value of the manuscript and brought forth the key factors that impact implementation of virtual wards at home.
